# Detection of Bacterial Activity via Reduction of Prussian Blue Thin Films as Probed by Raman Spectroscopy

**DOI:** 10.1021/acsomega.5c02367

**Published:** 2025-09-15

**Authors:** Bruna F. Baggio, Nga Tsing Tang, Cristiano Vicente, Jiaqi Luo, Andre A. Pasa, Rasmita Raval

**Affiliations:** † Open Innovation Hub for Antimicrobial Surfaces, Surface Science Research Centre, Department of Chemistry, 4591 University of Liverpool, Liverpool L69 3BX, United Kingdom; ‡ Postgraduate Program, Department of Physics, Federal University of Santa Catarina, Florianópolis 88040-900, Brazil

## Abstract

Biomarker-free and in situ detection of microbes and microbial biofilms is an important challenge. Prussian Blue (PB) has emerged as a potential material for a variety of biological applications. It is safe, sustainable, and also versatile, being employed as nanocarriers for drug delivery, antidotes for Cs and Tl poisoning, in photothermal therapy, and for the detection of live–dead bacteria. In this work, the ability of electrodeposited Prussian Blue (PB) thin films to detect bacterial activity was investigated using *Staphylococcus aureus* biofilm growth on the PB surface. The conversion of PB to Prussian White (PW), the reduced form of PB, due to the metabolic activity of *S. aureus*, was tracked using Raman microspectroscopy, enabling both time- and spatially resolved effects to be observed during biofilm growth. These results reveal that Raman spectroscopy can detect the onset of the reduction of the PB films after about 2.5 h of incubation time with *S. aureus* at 10^7^ CFU mL^–1^. In addition, localized Raman mapping of the PB surface reveals that the level of PB reduction varies across the surface in response to the local bacterial biofilm presence and activity. This work shows that Raman spectroscopy detection of the reduction of PB films by bacterial activity is a sensitive, direct, and nondestructive way of sensing the presence of live and metabolically active microbes in a localized way.

## Graphical abstract

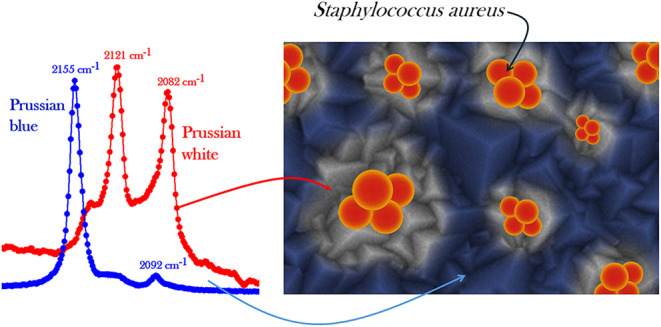

## 1 Introduction

Nondestructive, biomarker-free, and in situ detection of microbes and microbial biofilms is an important challenge across a number of applications, ranging from the detection of infection and biofouling to contamination issues in water and food security.^1,2^ There are different approaches adopted, depending on the analysis required.^3^ Methods such as counting colony forming units (CFUs) and live/dead quantitative polymerase chain reaction (qPCR) techniques provide quantitative evaluation of the number of cells present, but are destructive, laborious, and time-intensive,^4,5^ with qPCR also incurring significant cost. Alternatively, fluorescent dye-based approaches such as the Syto 9–Propidium Iodine test^6^ provide visualization of bacterial live–dead status. Approaches that measure bacterial metabolic activity^7,8^ are particularly interesting since they go beyond a viable cell counting or killing assessment, enabling a more nuanced evaluation of biofilm behavior and antimicrobial and antibiofilm interventions and technologies. Here, we study the promising indicator-detection system provided by electrodeposited thin films of Prussian Blue (PB), i.e., iron­(III) hexacyanoferrate­(II), which undergoes a visible color change when reduced by bacterial activity.^9–13^ Importantly, this system has the potential to be tracked via Raman spectroscopy due to the distinct vibrational signatures for the PB and PW phases, enabling clear and rapid detection with high spatial resolution. The spectral sensitivity to chemical changes also opens a promising future route toward quantification of bacterial activity, which is an underpinning need in standard microbial and biofilm test methods.^7,8^

PB exhibits a very distinctive blue color and is a compound with cubic crystalline structure and a general molecular formula AFe^III^[Fe^II^(CN)_6_], where A is an alkali metal. The alkali atoms balance the charge neutrality in the crystal, and the compound is known as PB. It is a mixed-valence complex with two distinct iron sites: Fe­(II) coordinated to carbon atoms and Fe­(III) coordinated to nitrogen atoms.

PB can accept electrons, and the electron transfer to the Fe cations coordinated to N atoms occurs simultaneously with the intercalation of A cations from the medium into interstitial sites in the lattice. This phase transition results in a colorless compound, A_2_Fe^II^[Fe^II^(CN)_6_], which is known as Prussian White (PW).^14^

The redox properties of PB have led to several applications such as sensors,^15^ magneto-optical devices,^16^ alkali metal-based batteries,^17–20^ and colorimetric, optical, and electrochemical detection of bacteria.^12,13,21,22^ PB is also a metabolic indicator allowing the detection of live bacteria^9–11^ as the compound can be reduced by living microorganisms. Bacterial metabolism instigates extracellular electron transport in microorganisms that leads to the transport of electrons out of the cells,^23,24^ and these electrons can then promote the reduction of the Fe­(III).^25,26^ This is a widely observed mechanism in organic matter degradation and nutrient cycling in soils and sediments.^27,28^ Figure 1 illustrates the reduction of PB to PW in the presence of the A^+^ cation in the medium. For the system studied here, it is considered that the phase transition involves the uptake of sodium (Na^+^) ions from the nutrient broth, enabling charge compensation in the crystalline structure of the material.

**Figure 1:**
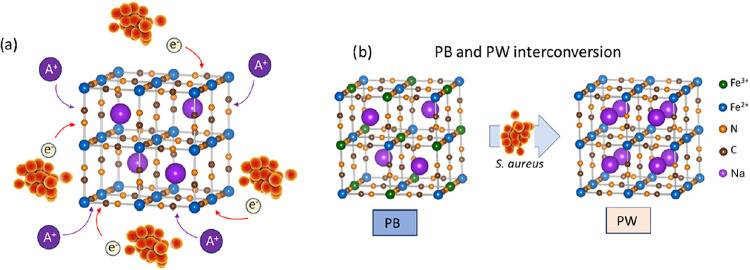
Schematics illustrating (a) charge-transfer processes occurring in a face-centered structure promoted by microorganisms that supply electrons to reduce the compound with the simultaneous uptake of A^+^ ions from the medium and (b) details of the phase transition from PB to PW arising from the reduction of Fe­(III) species, with the incorporation of Na^+^ ions into the interstitial sites.

A number of studies have shown that PB nanoparticles (PB-NPs) undergo phase transition to PW by the metabolism of both Gram-positive and Gram-negative bacteria^9–13,21,22^ with a complete color change reported after 40 h for PB-NPs embedded in textiles.^9^ In contrast, the use of thin films of PB for bacterial detection is little explored, despite the fact that highly uniform, crystalline, and low-defect PB thin films can be obtained through electrodeposition methods and have been widely studied for other technological applications.^19,29–31^ Bacterial detection using thin-film detection can be advantageous since it often presents a more rapid response.^10^ Furthermore, these films allow for the direct investigation of the behavior and activity of attached biofilms, enabling the real-time tracking of metabolic processes during biofilm growth. An early example of bacterial detection using PB thin films was demonstrated by Ferrer-Vilanova at.al,^10^ who studied *Escherichia coli* activity on PB thin films deposited on indium tin oxide-poly­(ethylene terephthalate) (ITO-PET) substrates. Subsequently, Ramasamy et al.^32^ and Psotta et al.^33^ used PB thin films electrodeposited onto screen-printed carbon electrodes (SPCEs) to monitor bacterial growth in blood cultures and to detect urinary tract infections (UTIs), respectively. In all of these cases, the PB-to-PW transition was monitored electrochemically. In this work, we have studied electrodeposited PB thin films and investigated whether the metabolic activity of Gram-positive *Staphylococcus aureus* (*S. aureus*) bacterial biofilms can be detected by following the PB-to-PW phase change using Raman microspectroscopy. The transition can be observed visually through the change of color; however, this approach has limitations in terms of time and length scales over which color changes can be discerned and quantified. Importantly, the PB-to-PW spectral changes occur in a part of the Raman spectrum that is free from signals arising directly from the biological entities and, therefore, can be tracked easily without background interference. Our work demonstrates that the Raman technique enables direct and nondestructive detection of the reduction of PB to PW with good sensitivity and, additionally, allows both time-resolved and spatially resolved changes to be obtained.

## 2 Experimental Section

### 2.1 Thin-Film Deposition

The PB thin films on Au substrates were prepared via electrochemical synthesis by a potentiodynamic technique, using an Autolab PGSTAT 302N electrochemical workstation. The electrochemical deposition was performed at room temperature in a conventional three-electrode cell, with Au/Cr/Si as the working electrode, platinum foil as the counter electrode, and a saturated calomel electrode (SCE) as the reference. The electrolyte for PB synthesis consisted of 0.25 mM FeCl_3_, 0.25 mM K_3_[Fe­(CN)_6_], 5 mM HCl, and 1 M KCl at pH 2.3.^29^ The substrates were prepared by an e-beam technique, consisting of layers of Au (50 nm) and Cr (5 nm) on Si-n(100) wafers. A circular area of 0.5 cm^2^ was delimited on the Au/Cr/Si surface for the electrodeposition of the PB films. The PB layers were grown by cycling the applied voltage from 0.70 to −0.25 V_SCE_, and the thickness is dependent on the scan rate and number of cycles. The expected electrochemical processes that promote sample growth are described as follows: first, the reduction reaction of Fe^3+^ + [Fe­(CN)_6_]^4–^ + 2K^+^ + e^–^ → K_2_Fe^II^[Fe^II^(CN)_6_], which leads to the formation of PW; this is followed by the oxidation reaction K_2_Fe^II^[Fe^II^(CN)_6_] – K^+^ – e^–^ → KFe^III^[Fe^II^(CN)_6_], leading to the formation of the PB layers.

### 2.2 Bacterial Studies

Bacterial culture preparation. *Staphylococcus aureus* (ATCC 6538P) was used in the work presented here. The overnight culture (ONC) was first prepared by inoculating a single colony of *S. aureus* from the solid media to 10 mL of nutrient broth (NB, OXOID Ltd., Cat. No.: CM0001) and incubated in the shaker incubator at 37 °C and 180 rpm for 18 h. The ONC was diluted to the desired concentration. 3 mL of the culture was added into each Petri dish with a PB sample placed at the bottom and incubated in a 37 °C incubator. At each time point, the PB sample was carefully taken out from the culture, placed under an optical microscope, and analyzed using Raman spectroscopy. The PB was then placed back in the same bacterial culture for further incubation for the next time point.

### 2.3 Characterization of the PB Thin Film and Bacterial Induced Changes

Scanning electron microscopy (SEM): A scanning electron microscope, SEM-FEG (JEOL JSM 7001F), was used for secondary electron imaging at 5 kV to determine the thin-film morphology and to image bacterial presence on the sample surface.

Raman spectroscopy: Raman spectra were obtained at room temperature with a Renishaw inVia micro-Raman spectrometer and WiRE 4.2 software. A laser excitation wavelength of 532 nm was used to characterize the PB and PW phases present under different conditions. Optical images of the surface were captured with the digital camera attached to the Raman microscope, and the PB film was studied as a function of bacterial incubation time. After each time point, the sample was placed under the optical microscope to observe visual changes before measuring Raman spectra. The results were unaffected whether samples were washed with phosphate-buffered saline (PBS) prior to the optical and Raman analyses, suggesting that an attached biofilm was formed on the PB thin film. Spatially resolved Raman data were obtained using a ×20 objective lens, enabling localized sampling from spots of 1.6 μm diameter, with a spectral resolution of 1 cm^–1^.

## 3 Results and Discussion

### 3.1 Potentiodynamic Growth of the PB Thin Film

PB layers were electrochemically synthesized on Au/Cr/Si substrates by the potentiodynamic method, as described in Section 2.1. This method leads to film growth occurring in two steps: at first the PW deposit is formed and the subsequent oxidation of the PW layers results in the PB film. Figure 2a shows a typical voltammogram recorded during PB growth. Well-defined reduction and oxidation peaks can be seen at about 0.17 and 0.28 V_SCE_, respectively. This voltammetric procedure is repeated many times in order to obtain films with hundreds of nanometers of thickness. Figure 2b displays an SEM image of the surface morphology of a film with a blue color grown after 50 cycles at a scan rate of 100 mV s^–1^, corresponding to a thickness of 200 nm, as measured with a profilometer. SEM data show that the resulting deposit is homogeneous, well-ordered, compact, and with pyramidal grains, characteristic of the ⟨111⟩ crystallographic growing direction.

**Figure 2:**
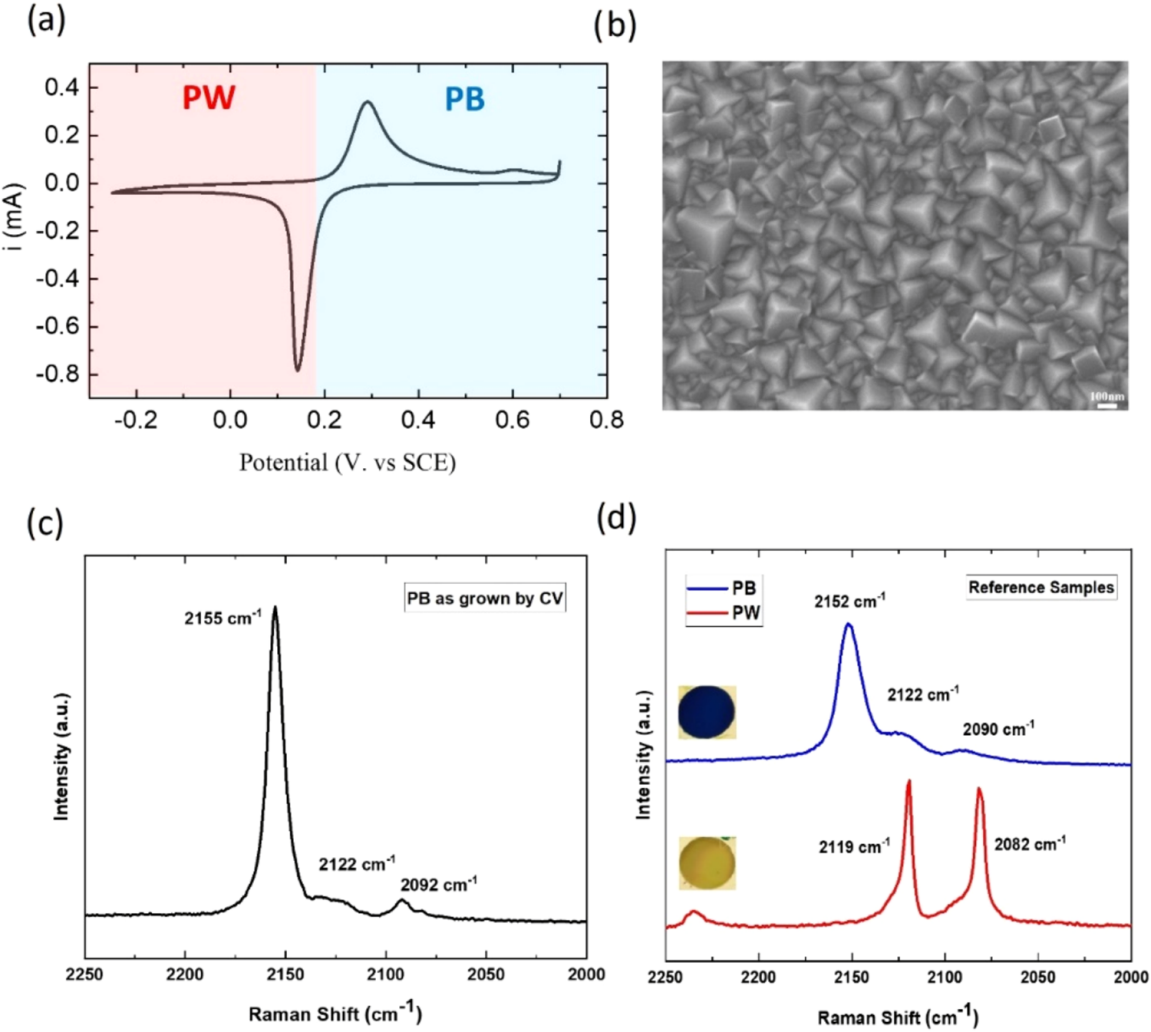
PB thin film on the Au/Cr/Si substrate. (a) Cyclic voltammetry (CV) during the electrochemical synthesis of the PB film by the potentiodynamic method. (b) SEM image of the surface morphology of the PB film as-grown. (c) Characteristic Raman spectrum of the PB sample prepared via potentiodynamic electrochemical synthesis. (d) Reference Raman spectra of PB and PW films prepared by the potentiostatic method.

The Raman spectrum of the synthesized PB film is shown in Figure 2c. In addition, Raman spectra of reference samples for PB and PW, prepared by the potentiostatic method,^29^ are also shown in Figure 2d. The Raman signature of each phase is distinctly different, which is reflected in spectral changes in the 2250–2000 cm^–1^ region arising from the CN vibrations, which are sensitive to the Fe oxidation state.^34,35^ In general, the higher the iron oxidation states, the stronger the Fe–CN σ-bond and the higher the frequency of the CN-stretching vibration.^36,37^ The PB phase shows an intense vibrational band at 2152 cm^–1^ and a much smaller peak at 2090 cm^–1^, respectively, assigned to the A_1g_ and E_g_ vibrations of the CN group in the PB phase [Fe­(II), Fe­(III)]. The PW reference spectrum, on the other hand, shows two intense peaks at around 2119 and 2082 cm^–1^ attributed to the corresponding A_1g_ and E_g_ vibrations of the CN group in the PW phase [Fe­(II), Fe­(II)].

### 3.2 Bacterial Metabolic Activity on PB Thin-Film Samples

#### 3.2.1 Color Change and Scanning Electron Microscopy

We first investigated whether the metabolic activity of the Gram-positive *S. aureus* bacteria could be observed visually on the PB thin films via its phase transition to PW and associated color change. PB electrodeposited on Au substrates was incubated with *S. aureus* at different initial bacterial concentrations. To ensure a reliable comparison between bacteria-exposed samples and control samples, the PB film on the Au/Cr/Si substrate was cleaved into three pieces, as illustrated in Figure 3a. The control sample C was placed in nutrient broth (NB), sample A1 was incubated with *S. aureus* at a concentration of 10^7^ CFU mL^–1^ in NB, and sample A2 with *S. aureus* at a concentration of 10^9^ CFU mL^–1^ in NB. After 5 h of incubation at 37 °C, the samples were taken out and inspected visually prior to the analysis via electron microscopy and Raman spectroscopy. The digital photographic images after incubation in Figure 3b show the control sample with the film retaining its blue color, while the films on samples A1 and A2 have suffered a significant loss of color.

**Figure 3:**
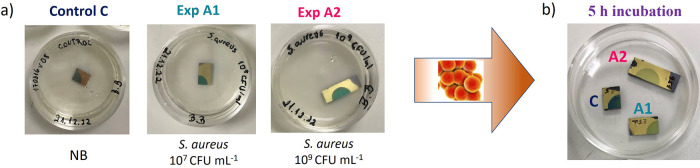
PB thin films on the Au/Cr/Si substrate after 5 h incubation with *S. aureus* at 37 °C. (a) Digital photos of the control sample C before being immersed in NB and samples A1 and A2 before incubation with *S. aureus* in NB at 10^7^ and 10^9^ CFU mL^–1^, respectively. (b) Digital photo of the samples after 5 h of incubation.

The color change data show that *S. aureus* activity does indeed lead to a PB-to-PW transition in the thin films. In order to obtain a microscopic view, SEM images of each type of sample after 5 h incubation are shown in Figure 4. For the control sample C, only the PB layer is observed, with the characteristic pyramid morphology being visualized at a higher magnification in Figure 4a. At the lowest magnification, some deposits of NB are visible on top of the film. For the bacteria-exposed samples, formation of early biofilm stage *S. aureus* clusters on the PB thin films is evident. On sample A1, smaller clusters are observed for the lower bacterial seeding concentration of 10^7^ CFU mL^–1^ (Figure 4b), while on sample A2, larger clusters of *S. aureus* are present at the higher seeding concentration of 10^9^ CFU mL^–1^ (Figure 4c). In addition, it is noted that the morphology of the PB layers remains unaltered in areas of the film that are unpopulated by bacteria, suggesting that the PB thin film does not undergo a transition in the absence of the biofilm. The SEM images also demonstrate how the effect of bacteria at a local level is captured in a microscopic technique, compared to the macroscopic visual technique that simply shows a general loss of the blue color.

**Figure 4:**
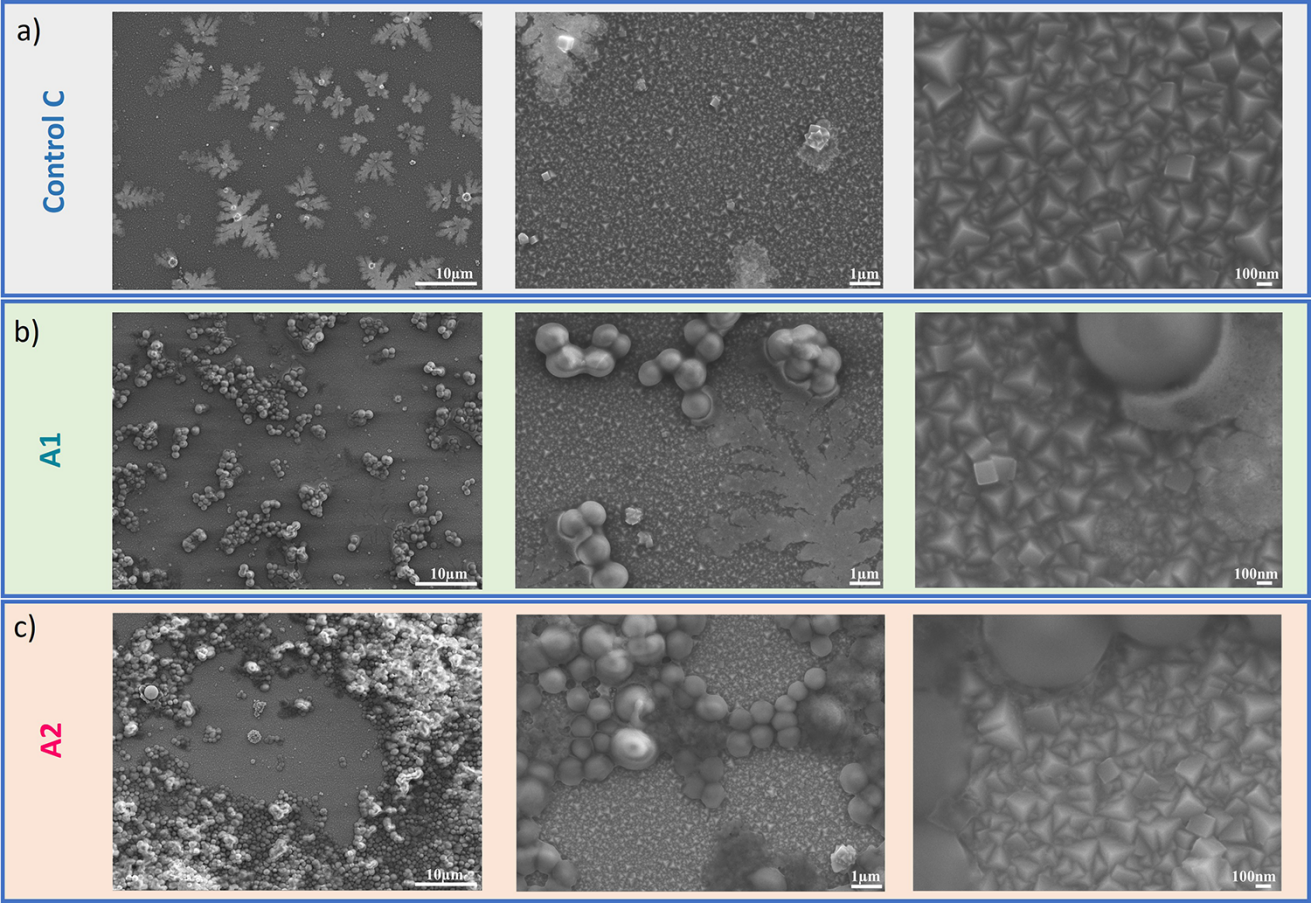
SEM images of PB surfaces after 5 h incubation at 37 °C. (a) Control sample C (PB sample in nutrition broth), (b) sample A1 (PB incubated with *S. aureus* at 10^7^ CFU mL^–1^ in NB), and (c) sample A2 (PB incubated with *S. aureus* at 10^9^ CFU mL^–1^). The scale bars are 10 μm, 1 μm, and 100 nm for the first, second, and third columns, respectively.

#### 3.2.2 Raman Spectroscopy

Considering that each phase of the film gives rise to a distinct Raman spectrum, as shown in Figure 2d, the next step was to investigate whether the PB-to-PW transition arising from the activity of the *S. aureus* biofilm could be detected using Raman spectroscopy. Optical microscopy images obtained after 5 h of incubation in NB (control sample C) and with *S. aureus* at two different concentrations in NB (Samples A1 and A2) are shown in Figure 5 (top panel). The corresponding representative Raman spectrum from each sample is displayed in the bottom panel of Figure 5. The optical image of the control sample displays a dark blue and uniform surface typical of PB. However, samples exposed to bacteria show surfaces with both dark and light areas, with the preponderance of the light regions increasing with the higher cell seeding concentration, as seen for sample A2. The changes observed in the optical images are in general agreement with the digital photographic images shown in Figure 3.

**Figure 5:**
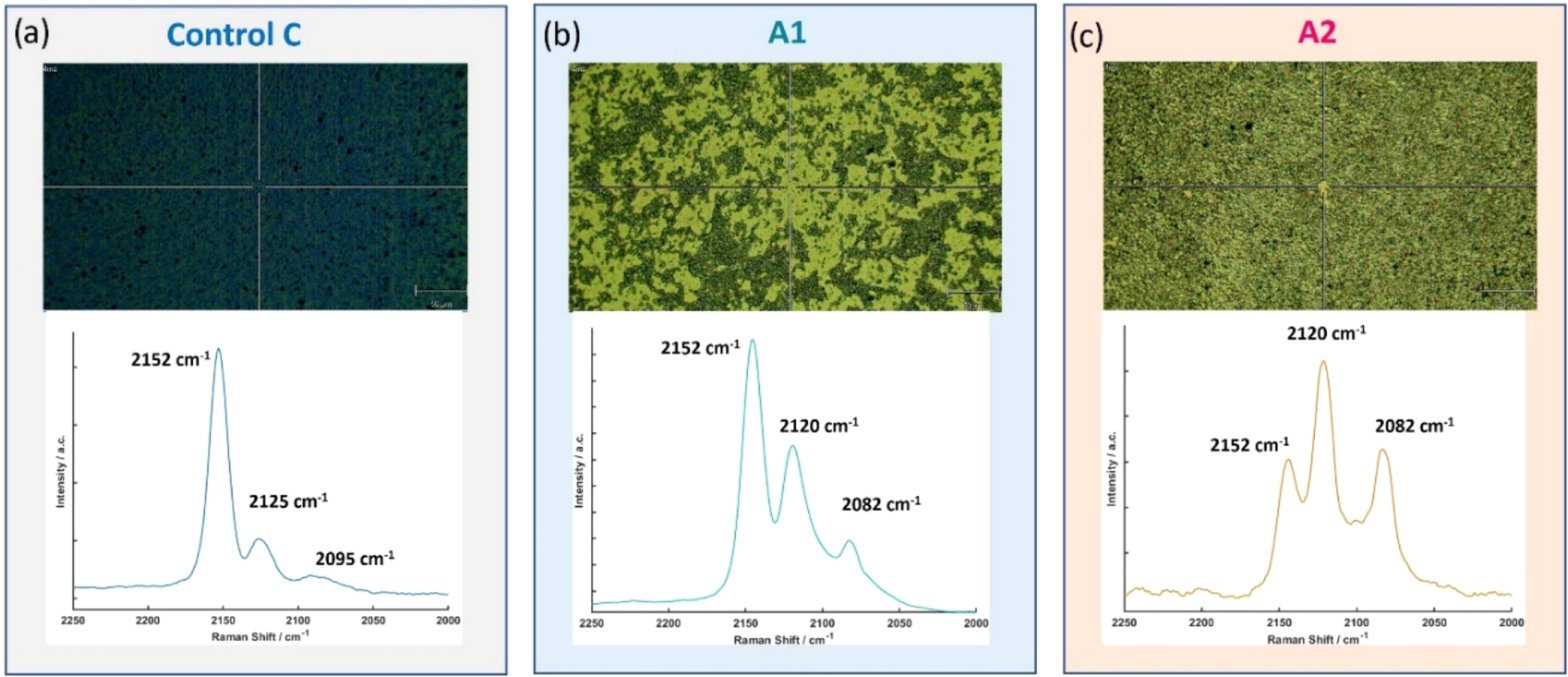
PB thin films on the Au/Cr/Si substrate after 5 h incubation at 37 °C with *S. aureus*. (a) Control sample C (nutrition broth) and experiment samples (b) A1 (*S. aureus* at 10^7^ CFU mL^–1^) and (c) A2 (*S. aureus* at 10^9^ CFU mL^–1^). Top: Optical images. Bottom: Raman spectra.

The Raman spectra also showed noticeable differences among the three samples. For the control sample C in Figure 5a, the characteristic spectrum for the as-grown PB film is obtained, similar to Figure 2c, with a signature strong band at 2152 cm^–1^ and lower contributions at 2125 and 2095 cm^–1^. However, spectral changes are observed upon exposure to the bacteria, with samples A1 and A2 showing the emergence of strong bands at 2120 and 2082 cm^–1^, indicating that Fe­(III) ions are reduced to Fe­(II) and that the PB is reduced to PW.^34–37^ The transition in Raman spectra from the control sample to samples A1 and A2 shows that the phase transition occurs in a stepwise manner. The peaks due to PW grow in relative intensity to PB peaks as the bacterial concentration is increased, inducing greater biofilm growth, as depicted in the SEM data in Figure 4.

As described earlier, the difference between PB and PW is that the C–N ligands are coordinated to Fe ions with different oxidation states, and the transition occurs with the concomitant incorporation of an alkali metal to compensate for the negative charge of the electron provided by the bacteria from their metabolic activities. Since the nutrition broth contains NaCl, it is considered that the uptake of Na^+^ cations provides the charge compensation, resulting in the PW phase with chemical formula NaKFe^II^[Fe^II^(CN)_6_].

Given the ability of Raman spectroscopy to detect the effect of the presence and metabolic activity of bacteria on the PB films, we undertook time-resolved studies to investigate the onset of metabolic activity in the system. Figure 6 shows the change of the Raman spectra as a function of incubation time with *S. aureus* at a 10^7^ CFU mL^–1^ seeding concentration in NB. To obtain sequential Raman measurements, the samples were removed from the *S. aureus* culture at specific time points to obtain the Raman data before being returned to continue incubation in the culture. The results show that bacterial activity leads to an increase in the intensity of the Raman peak at 2120 cm^–1^ over the initial 8 h period, a change which is already discernible after early incubation times between 1.5 and 2.5 h (see Figure 6). The Raman changes at the early time points suggest that the PB film is able to detect the metabolic activity increase that occurs shortly after the initial lag phase of bacterial growth. As the incubation time is increased to 8 h, the peak characteristic for PB at around 2150 cm^–1^ decreases in intensity, while the peak at 2120 cm^–1^, associated with PW, increases in intensity. For long incubation times, up to 24 h, the spectrum is dominated by two peaks at 2119 and 2082 cm^–1^ representing the PW phase, in agreement with the Raman signature from the PW reference sample (Figure 2d). Systematic visual checks of the sample surface with bare eyes showed the blue color fading after incubation times of >5 h. We note that the Raman spectra of the control sample remained unchanged after 7 h of incubation in NB (see Figure S1).

**Figure 6:**
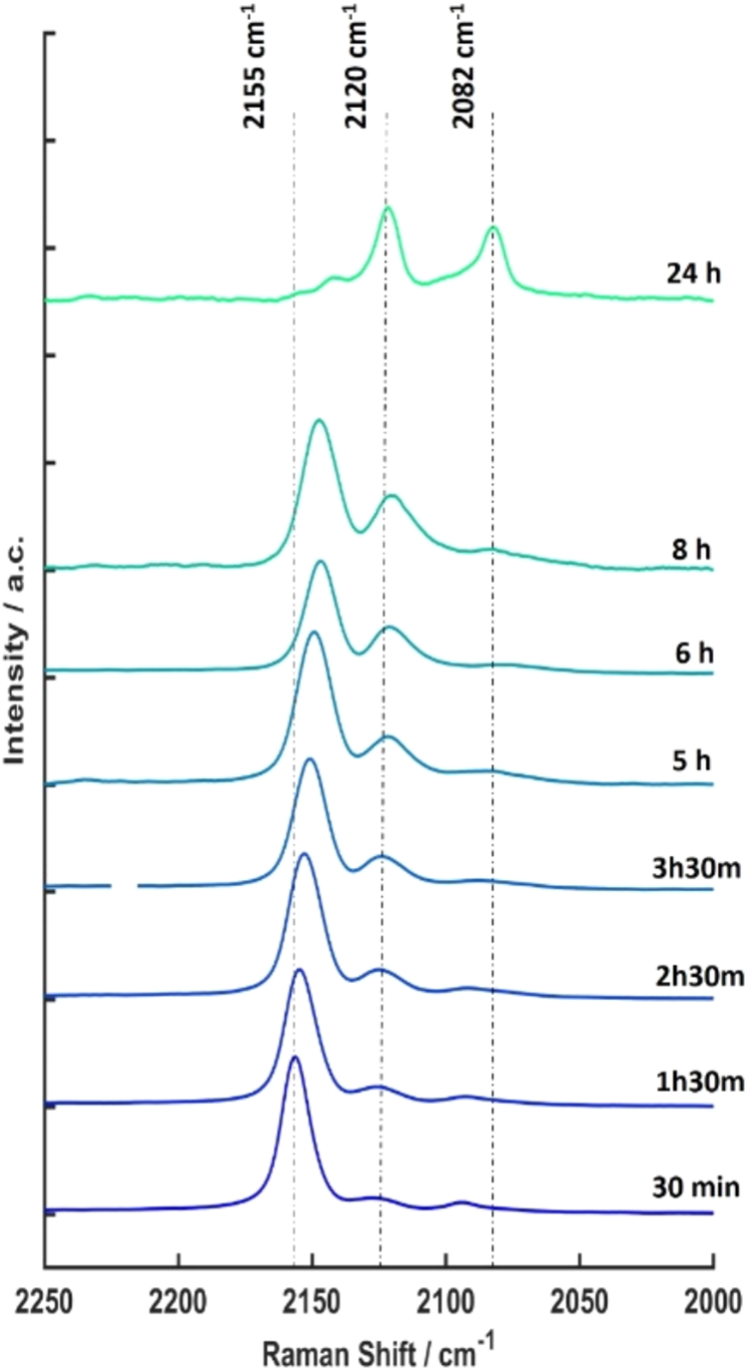
Time dependence of Raman spectra for a PB thin-film sample incubated with *S. aureus* in an NB suspension with an initial seeding concentration of 10^7^ CFU mL^–1^.

The mottled color changes observed under the optical microscope (Figure 5) suggest that the PB-to-PW transition occurs at a local level, presumably dependent on the level of the biofilm present locally at the surface. In order to investigate whether local film responses can be detected by Raman spectroscopy, spatially resolved data were collected across the surface. Figure 7a shows the optical image of a sample incubated for 24 h with *S. aureus* in NB with a seeding concentration of 10^7^ CFU mL^–1^. Individual Raman spectra with a good signal-to-noise ratio were obtained from 1.6 μm diameter spots with a step size of 10 mm in a grid across a 150 mm × 200 mm area depicted by the box on the optical image. This Raman mapping reveals that three significant PB/PW peaks at 2140, 2120, and 2083 cm^–1^ are observed with different relative intensities across the points sampled at the surface, indicating distinct stages of the sample phase change at the local level (see Figure S2). A heatmap showing the intensity ratio between the 2120 and 2140 cm^–1^ peaks is plotted in Figure 7b, which provides an indicator of the PW/PB ratio. In the lighter blue area in Figure 7a, region 1, higher values of the PW/PB ratio are obtained corresponding to the predominance of the PW phase, while, in region 2, the lower values are observed due to the predominance of the PB phase. Broadly, this result is consistent with a greater bacterial activity in region 1 compared to region 2. Importantly, the fluctuation of the PW/PB ratio within each sampled spot confirms that the reduction of PB to PW occurs at the local level in response to the local bacterial activity from the attached biofilm. These general conclusions are also supported when K-means clustering is applied to the Raman mapping data (Figure S5).

**Figure 7:**
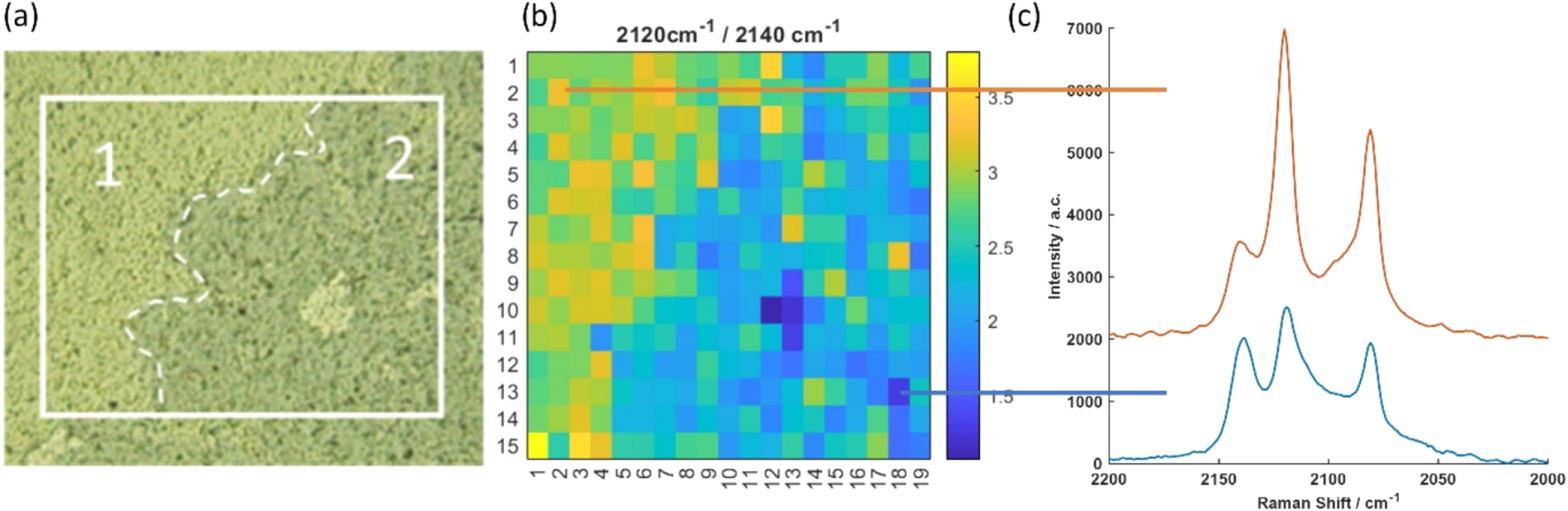
Raman map of the sample incubated with *S. aureus* at a bacteria seeding concentration of 10^7^ CFU mL^–1^ for 24 h. (a) Optical microscopy image of the area under investigation, with Raman spectra obtained via sampling 1.6 μm diameter spots across the selected sample area depicted within the white square. (b) Heatmap showing the fluctuation in the ratio of the 2120/2140 cm^–1^ peaks across the surface. (c) Examples of local Raman spectra from two selected spots, one showing a higher PW/PB ratio (in red) and one showing a lower PW/PB ratio (in blue).

## 4 Conclusions

This work showed that Prussian Blue (PB) thin films electrodeposited by the potentiodynamic method can detect the metabolic activity of *S. aureus* bacterial biofilms, which convert PB to its reduced form, Prussian White (PW). The transition from PB to PW induced by the bacteria was followed by the nondestructive, biomarker-free technique of Raman spectroscopy, which allowed time-dependent and spatially resolved changes to be observed during biofilm growth. Raman spectroscopy detected the onset of the reduction of the PB films after 2.5 h of incubation time with *S. aureus* seeded at 10^7^ CFU mL^–1^ in nutrient broth. Furthermore, Raman microscopy mapping revealed that the PB-to-PW reduction is induced at a local level, in response to the bacterial biofilm presence and activity. Thus, Raman detection of the reduction of PB films by bacterial activity is a sensitive and direct way of sensing the presence of live and metabolically active microbes. In addition, the ability to obtain Raman data from micron-level spot sizes shows the potential of this approach to spatially address the biological behavior and impact of biofilms.

The PB-to-PW spectral changes occur in a part of the Raman spectrum that is free from Raman signals arising directly from the ‘fingerprint’ spectra of biological entities. Therefore, bacterial activity can be tracked at good sensitivity without background interference, opening up the ability to map the dynamic metabolic behavior of biofilms as a function of surface attachment, growth, environment, and interventions, which are important and challenging topics in biofilm research.^2,38–40^ Specifically, the ability to obtain this information at high spatial resolution would enable metabolic gradients and interspecies effects to be monitored.

Although the current work was conducted on research-grade Raman instrumentation, the method has the potential to be translated to point-of-use applications using hand-held Raman devices, which are increasingly being deployed in the field, for example, in security, forensics, quality assurance in pharmaceuticals and drink industries, medical applications, space science, etc.^41–44^ In addition, electrodeposition technology has been successfully utilized across the electronics, semiconductor, and corrosion protection industries,^45^ providing a viable route to delivering low-cost and high-quality PB films.^19,29,30^

Finally, we note that our work represents a proof-of-concept study and is qualitative at present. However, the clear spectral changes associated with the PB-to-PW transition induced by microbial activity open up routes toward quantitative measurements of metabolic activity.

Standard validation in biofilm research is an active current topic that is being debated internationally and requires significant effort across multiple laboratories to establish quantitative benchmarks for methodological validation.^3–8^ In particular, the validation of our approach will need to be undertaken beyond CFU counts, since it is the metabolic activity that needs to be cross-validated rather than the number of viable cells. This is a significant research challenge and will require validation using other methods that assess comparable metabolic activity,^46,47^ many of which are also at early stages of validation. Ultimately, cross-validation will be required at the local level since biofilms possess gradients of metabolic activity. This will require localized nondestructive spectroscopic, dye, and electrochemical probes that are currently being explored.^48^ These are frontier questions in the biofilm field, and progress will take concerted effort by multiple research groups.

## Supplementary Material



## Notes

Raman spectroscopy of control samples—PB thin film immersed in NB—measured at different time points; Raman map showing the spectra of the PB surface incubated for 24 h with *S. aureus* in NB, corresponding to the area shown in Figure 7; Raman Spectroscopy of a Visually Homogeneous Area; T-test of Independent Samples; K-means clustering applied to Raman data. (PDF)

## §

Brazilian Nanotechnology National Laboratory, Brazilian Center for Research in Energy and Materials, 13083-100 Campinas, SP, Brazil
