# Supplementary material for: Detection of Bacterial Activity via Reduction of Prussian Blue Thin Films as Probed by Raman Spectroscopy
Source: ACS Omega. 2025 Sep 15;10(38):43510–8. doi: 10.1021/acsomega.5c02367 (PMC12489613; doi:10.1021/acsomega.5c02367)
Supplement: Supplementary file 1 [file ao5c02367_si_001.pdf]

Detection of Bacterial Activity via Reduction of Prussian Blue Thin Films as Probed by Raman Spectroscopy

Bruna F. Baggio[a,b], Nga Tsing Tang[a], Cristiano Vicente[b], Jiaqi Luo[a], Andre A. Pasa[b], Rasmita Raval[a*]

[a] Open Innovation Hub for Antimicrobial Surfaces, Surface Science Research Centre, Department of Chemistry, University of Liverpool, L69 3BX, United Kingdom

[b] Postgraduate Program, Department of Physics, Federal University of Santa Catarina, 88040-900, Brazil

Corresponding Author: R. Raval (raval@liverpool.ac.uk)

Supporting Information

S1. Raman Spectroscopy of Control Samples at Different Timepoints

PB thin films on Au were placed in NB as control samples. The Raman spectra showed no evidence of the PW phase formation as expected.

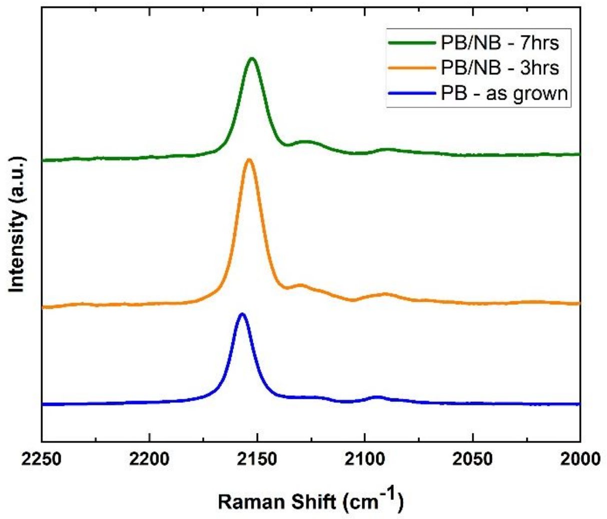

Figure S1: Raman Spectra of a control PB sample incubated in NB after specific timepoints.

S2. Raman Spectroscopy: Mapping

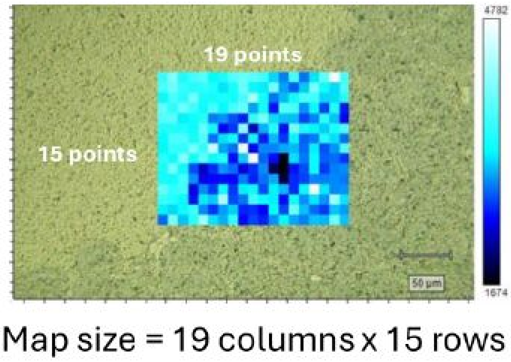

* Spectra from each point of the map, from left to right, top to bottom:

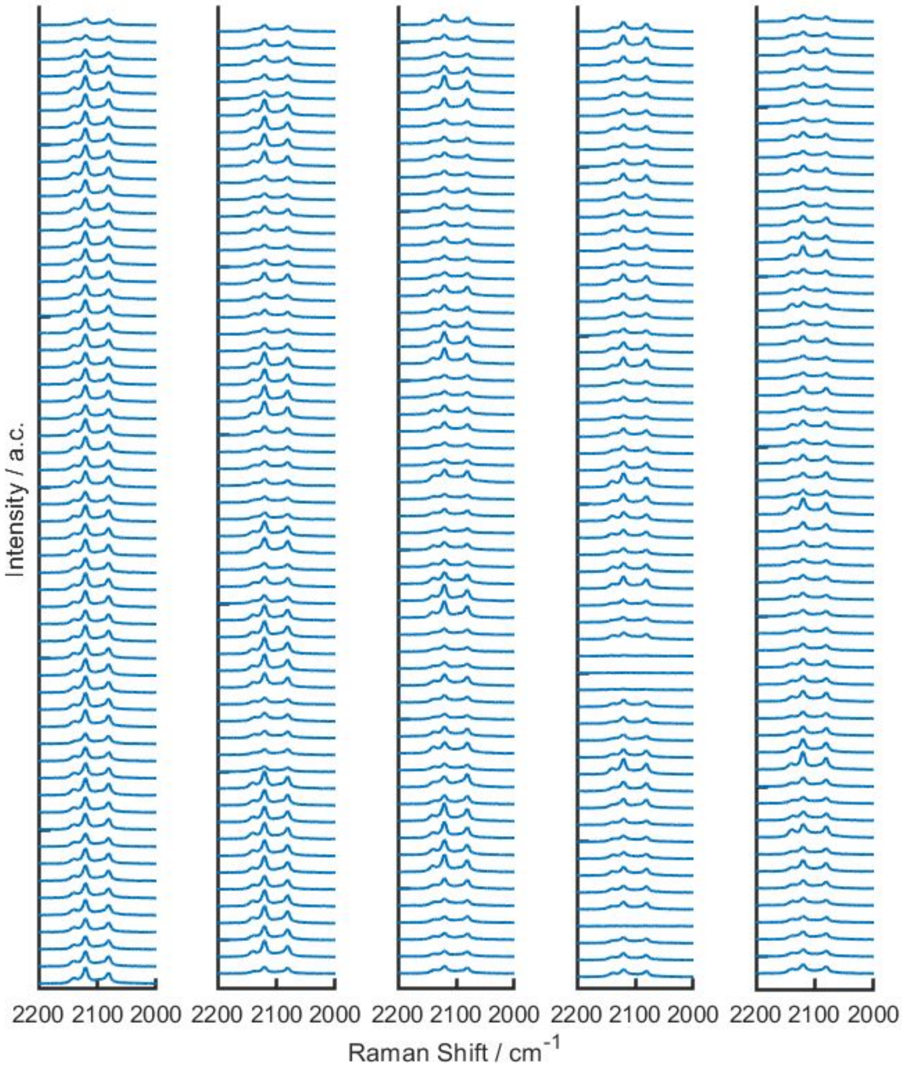

Figure S2: The Raman map of the surface of a PB sample incubated for 24 hrs with *S. aureus* in NB with an initial bacteria seeding concentration of 10^7 CFU mL^{-1}.

S3. Raman Spectroscopy of a Visually Homogeneous Area

Despite the region appearing visually homogeneous under optical microscopy, micro-Raman spectroscopy reveals distinct spectral variations within a 2 μm area, indicating different transitional phase stages of the surface. This highlights the sensitivity of Raman technique and its potential for probing localized biochemical activity within biofilms.

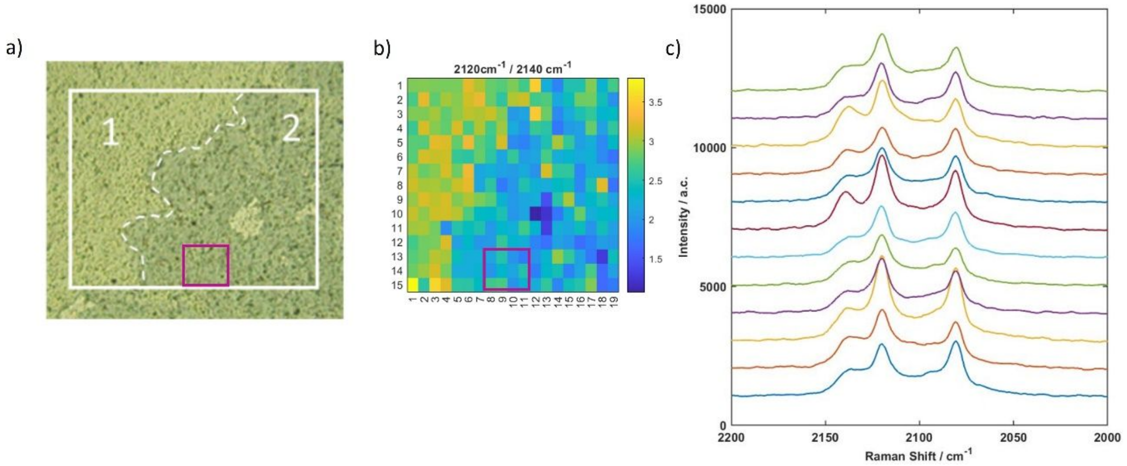

Figure S3. PB/PW-modified surface colonized by bacterial biofilm. a) Optical microscopy image. b) Spatially resolved Raman mapping showing the ratio of the 2120 to 2140 cm^{-1} peaks across the surface. c) Curves for different points in the selected area in b), representing the intermediate phases.

S4. T-test on T00 and T24 of Independent Samples

A t-test was used to assess the variation across time points (T00 = 0 h and T24 = 24 h) for several samples. A MATLAB built-in two-sample t-test function was applied to the intensities of the three Raman peaks associated with the PB to PW transition. Control T00 samples (PB+NB) of three replicates were selected (20 data points from 3 replicates) as well as T24 experimental samples (PB+NB+Bacteria) combining data sets from three replicates (69 data points from 4 replicates).

The results are presented in Table S4, with reference to the null hypothesis (H_0). All statistical tests were conducted at a 5% significance level. Significance is denoted as follows: *: $p < 0.05$ (statistically significant), **: $p < 0.01$ (highly significant), and ***: $p < 0.001$ (very highly significant).

The change observed from T00 to T24 is considered very highly significant based on the t-test results.

Table S1. Results of t-test

w/ SVN	Peak 2152	Peak 2120	Peak 2080
Null Hypothesis h0	rejected	rejected	rejected
P-value	< 0.001***	< 0.001***	< 0.001***

S5. k-means Clustering Analysis on the Raman Map

k-means clustering analysis was applied to the Raman mapping data (Fig 7 of the manuscript and associated Raman spectra SI Fig S2) using the MATLAB built-in algorithm with k = 2, 3 and 4, shown below. These cluster maps align with our general observation that region 1 on the left of the sample has higher values of the PW/PB ratio, corresponding to the predominance of the PW phase, while region 2 in the middle and right of the sample shows a lower PW/PB ratio due to the predominance of the PB phase. Since the PW/PB surface is undergoing transitional changes, defining the optimal number of clusters (k) for area classification is challenging. However, when k is set to 3 or 4, fluctuations of the PW/PB ratio within each region become apparent, indicating that the reduction of PB to PW occurs locally in response to bacterial activity from the attached biofilm. Notably, the high signal-to-noise ratio in the clean region of the Raman spectrum ensures that direct band ratio plotting provides a clear and reliable representation of PB/PW fluctuations across the sample.

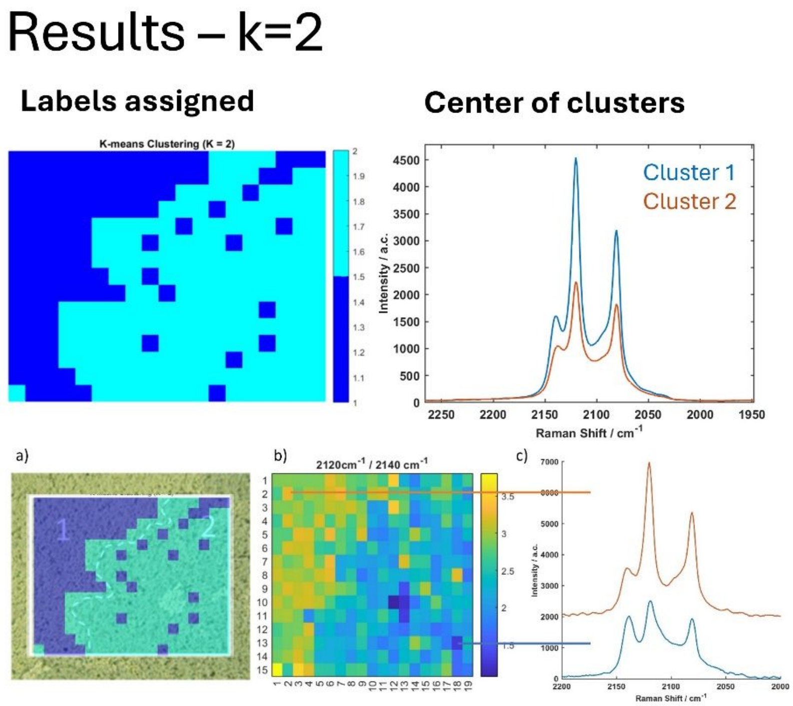

Figure S5. k-mean clustering results of the Raman map data when k = 2

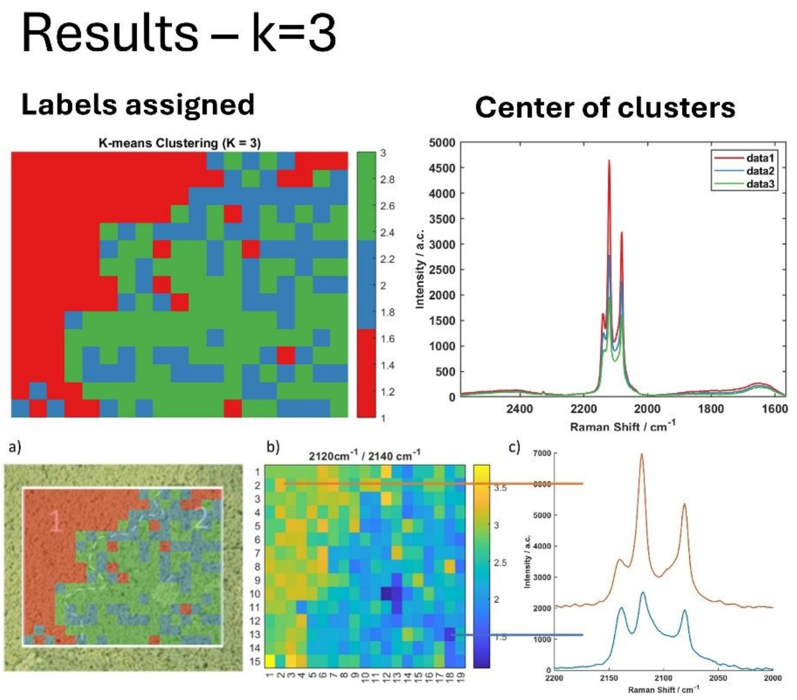

Figure S6. k-mean clustering results of the Raman map data when k = 3.

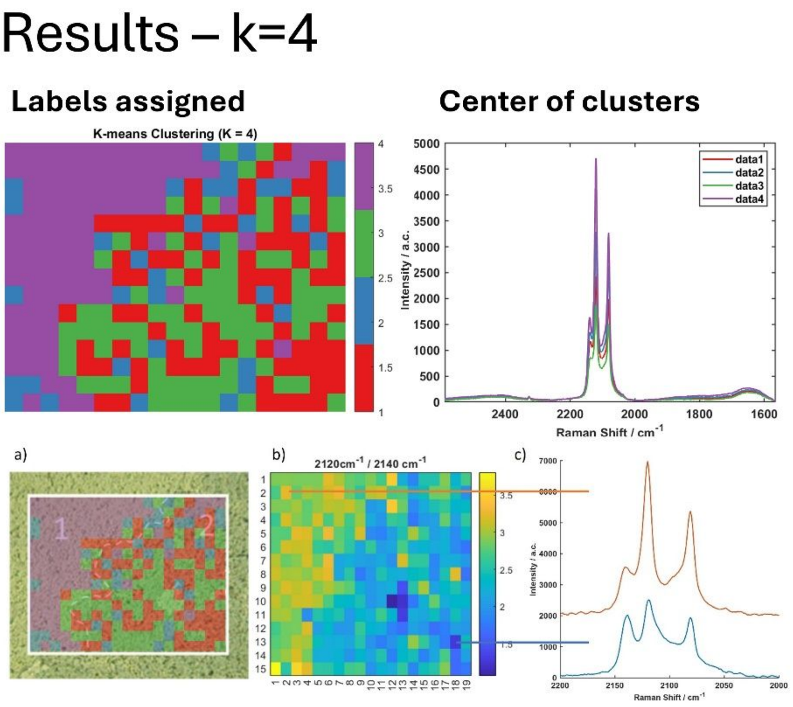

Figure S7. k-mean clustering results of the Raman map data when k = 4.
